# Public health insurance and maternal health care utilization in india: evidence from the 2005–2012 mothers’ cohort data

**DOI:** 10.1186/s12884-022-04441-4

**Published:** 2022-02-25

**Authors:** Tesfaye Alemayehu Gebremedhin, Itismita Mohanty, Theo Niyonsenga

**Affiliations:** 1 Faculty of Business, Government and Law, University of Canberra, Australian Capital Territory, Canberra, 2617 Australia; 2 Health Research Institute, Faculty of Health, University of Canberra, Australian Capital Territory, Canberra, 2617 Australia

**Keywords:** Maternal and child health care service utilization, Maternal autonomy and empowerment, Health insurance coverage, Public and private insurance

## Abstract

**Background:**

The introduction of Janani Suraksha Yojana (JSY) in India, a conditional cash transfer program which incentivized women to deliver at institutions, resulted in a significant increase in institutional births. Another major health policy reform, which could have affected maternal and child health care (MCH) utilization, was the public health insurance scheme (RSBY) launched in 2008. However, there is a noticeable lack of studies that examine how RSBY had impacted on MCH utilization in India. We used data from a cohort of mothers whose delivery had been captured in both the 2005 and 2011/12 rounds of the Indian Human Development Survey (IHDS) to study the impact of health insurance (in particular, the public insurance scheme versus private insurance) on MCH access. We also investigated whether maternal empowerment was a significant correlate that affects MCH utilization.

**Methods:**

We used the multilevel mixed-effects ordered logistic regression model to account for the clustered nature of our data. We derived indexes for women’s empowerment using Principal component analysis (PCA) technique applied to various indicators of women’s autonomy and socio-economic status.

**Results:**

Our results indicated that the odds of mothers’ MCH utilization levels vary by district, community and mother over time. The effect of the public insurance scheme (RSBY) on MCH utilization was not as strong as privately available insurance. However, health insurance was only significant in models that did not control for household and mother level predictors. Our findings indicated that maternal empowerment indicators – in particular, maternal ability to go out of the house and complete chores and economic empowerment—were associated with higher utilization of MCH services. Among control variables, maternal age and education were significant correlates that increase MCH service utilization over time. Household wealth quintile was another significant factor with mothers belonging to upper quintiles more likely to access and utilize MCH services.

**Conclusions:**

Change in women’s and societal attitude towards maternal care may have played a significant role in increasing MCH utilization over the study period. There might be a need to increase the coverage of the public insurance scheme given the finding that it was less effective in increasing MCH utilization. Importantly, policies that aim to improve health services for women need to take maternal autonomy and empowerment into consideration.

**Supplementary Information:**

The online version contains supplementary material available at 10.1186/s12884-022-04441-4.

## Introduction

India has made significant progress in reducing maternal mortality rate, which decreased from 370 to 145 per 100,000 live births between 2000 and 2017 [1]. This remarkable achievement can be attributed to the country’s concerted effort to increase women’s access to maternal health services, in particular the initiative to increase institutional births [2]. Institutional births rose from 35 percent in 2005 to 79 percent in 2016 [3]. However, the rate of maternal mortality decline was inadequate to meet the global Millennium Development Goal (MDG) target and India is still a significant contributor to global maternal deaths.

The renewed commitment to reduce maternal and infant mortality rates under the Sustainable Development Goals (SDG) and the amount of resources the government of India is investing to improve maternal and child health outcomes underscore the need to understand the dynamics of maternal and child health care utilization beyond simply focusing on institutional births. Encouraging women to give birth in health facilities has been an important global strategy to reduce maternal and perinatal deaths in low- and middle-income countries [4–6]. However, pregnant women require professional attention not only during the period of delivery but throughout the pregnancy and postnatal periods.

The Indian government in 2005 rolled out a conditional cash transfer program – known as Janani Suraksha Yojana (JSY)—to increase pregnant mothers’ access to maternal and child healthcare services [7]. Under the JSY program, pregnant women were given cash incentives to deliver in institutions and female community health workers (known as Accredited Social Health Activists (ASHA)) were paid to facilitate antenatal checkups, immunization of newborn babies and postnatal visits [8]. The program was designed to address inequality in access to maternal and child health care by mitigating financial barriers that thwarted poor women from accessing institutional care [9]. Several studies evaluating the JSY program had concluded that the program had been successful in increasing institutional births in India [10, 11]. However, there is evidence that inequality in institutional births has persisted [9] and recipients of JSY still incurred a significant out-of-pocket expenditure on maternal care [5, 8, 12].

Given that out-of-pocket payments to finance healthcare could be catastrophic to poor households [12, 13], in 2008 the Indian government initiated a publicly funded health insurance scheme -known as Rashtriya Swasthya Bima Yojana (RSBY) [14]. RSBY aims to protect poor Indian households (Below the Poverty Line (BPL)) from financial risks due to hospitalization and covers secondary inpatient care provided at community health centers, district hospitals, and medical colleges [14]. RSBY also covers maternity benefits and all expenses related to delivery at the hospital but excludes prenatal expenses. The maternity benefits included in the scheme may encourage more women to take-up maternal and health services. Moreover, if the scheme reduces the household overall out-of-pocket expenditure on healthcare, then savings made would help pregnant women (enrolled in the program) to cover expenses related to antenatal care and other MCH services. However, the effect of the public health insurance (RSBY) on MCH service utilization has not been investigated.

The wider literature also showed that giving women more decision-making power in the household leads to better educational and health outcomes for children [15–20]. Similarly, we would expect empowered women to utilize more MCH services given their role as primary caregivers of children. However, women continue to have little household decision-making authority in many developing countries including India.

Our study investigated if the introduction of RSBY improved the overall utilization of maternal and child healthcare (MCH) services using the 2005 and 2011–2012 rounds of the Indian Human Development Survey (IHDS). The study also investigated potential relationships between MCH utilization and women empowerment. It is important to study how MCH utilization has changed over time in India, especially in the era when the Indian government has invested considerable resources in the sector.

## Data and Methodology

### Data source: the 2005 and 2011–12 survey rounds of the Indian human development survey

The Indian Human Development Survey (IHDS) is a nationally representative survey covering 41,554 households in 2005 and 42,152 households in 2011–12. There were a panel of 40,018 households surveyed in both 2005 and 2011–12 rounds [21, 22]. The surveys collected information on a range of indicators including household and family structure, consumption and standard of living, health and gender relations, the status of women in the household such as their freedom of mobility and involvement in household decision making. The dataset provides a unique opportunity to study the dynamics of maternal and child health care utilization in India due to its longitudinal structure. Detailed information about survey sample design and implementation are contained in Desai et al. [23].

For our analysis, we identified mothers who had given birth in the two survey periods covered in the IHDS. In particular, the women’s questionnaire had detailed questions about children born in the few years preceding the two surveys. For example, the 2005 survey had questions about children born ‘since January 2000’, while the 2011–12 survey asked similar questions of children born ‘since January 2005’. The type of questions asked in the survey pertained to place and type of delivery, whether the mother had antenatal and postnatal checkups, complications during the pregnancy, breastfeeding and immunization, presence and type of insurance and so forth [23]. We were able to identify 4,289 mothers who had given birth both in the 2000–2005 and 2005–2012 periods, allowing us to compare changes in maternal and health care service utilization across the two survey rounds.

### Outcome measure: maternal and child health care utilization

Our study combined three indicators of maternal and child health outcomes – institutional births, antenatal and postnatal checkups. The first indicator used in our study to measure maternal and child health care (MCH) utilization was whether the mother had delivered at an institution. The relevant question in the IHDS survey questionnaire asked mothers *‘….at what kind of place…’* they had delivered, and they had to choose between the following answers: *i*) Government hospital or clinic; *ii*) private nursing home; *iii*) Home; and *iv*) others. We considered a mother to have had institutional birth if she had delivered at government hospital or clinic or private nursing home. The second indicator was the number of antenatal checkups the mothers had received during their pregnancy. The aim of antenatal care (ANC) is to identify and manage obstetric complications. Other services such as tetanus toxoid immunization and intermittent preventative treatment for malaria during pregnancy (IPTp) are administered during ANC visits. ANC also presents with the opportunity to promote institutional delivery and healthy practices such as breastfeeding, early postnatal care and birth spacing [24]. WHO [25] guidelines on maternal and neonatal care suggested that pregnant women should receive at least four antenatal care (ANC) visits prior to delivery. Therefore, in our study, we considered four to be the minimum number of ANC visits pregnant women should receive. The third indicator was whether the mother had postnatal checkups (for herself and her child). The postnatal period is crucial for the lives of mothers and their newborn babies since lack of appropriate care during this period could result in serious illness or even death [26]. For our study purposes, we used the question in IHDS that asked mothers if *‘…a doctor or other health professional…’* had checked their health or that of their baby in the 2-month period after the delivery. Having had a postnatal checkup completed by a doctor or health professional was considered as appropriate indicator of MCH utilization [25].

Our categorical outcome variable – MCH utilization- was coded as zero – if a mother does not meet any of the three indicators – i.e. she did not deliver at an institution; she had less than four ANC visits; and didn’t receive any postnatal checkup. The MCH utilization was coded as: 1 – if the mother meets the best outcome in only one of the indicators, and 2 – if she meets the best outcome in two of the indicators, and 3 – if the mother meets the best outcome in all three indicators above, that is, she had delivered at an institution; she had received 4 or more ANC visits; and she had postnatal checkups. Therefore, our categorical outcome variable is ordered in nature and ranges from 0 (where the mother does poorly under all three indicators) to 3 (where the mother meets the best outcome for all three indicators).

### Exposure variable

The insurance and financial assistance schemes available in India in 2005 included private insurance schemes, various public sector schemes offered to employees, specific state government insurance schemes offered to residents in their states, and other schemes focussed on specific groups (such as JSY for pregnant women). The RSBY was added on top of these various schemes in 2008, between the 2005 and 2011–12 rounds of the IHDS.

Our main exposure variable was the household health insurance coverage status, assessing whether the household had benefitted from health insurance in the five years prior to the survey [22]. Since the publicly funded health insurance scheme was not rolled out until 2008, those women whose delivery was recorded in the 2005 round had not benefitted from RSBY. However, the 2005 IHDS data asked if surveyed households had availed themselves of private health insurance. Thus, our main exposure variable health insurance captured only private health insurance in 2005 but included both private and public insurance in 2011–12. Our analysis would show if health insurance, in general, had been a significant factor in increasing women’s MCH utilization and, in particular, if the public insurance funded by the government had been effective compared to private health insurance.

### Confounding variables

Our study controlled for a range of potential confounding variables, which were believed to affect women’s utilization of MCH services in the wider literature. We controlled for individual maternal variables such as maternal age [27, 28] and maternal educational attainment [29–31]. We also controlled for household level variables including household size and number of children [32]. Household economic status, measured using quintile of consumption per capita as well as by the below poverty line status of households,  was also adjusted for.

To examine the impact of maternal empowerment on MCH utilization, we constructed indexes to capture women empowerment using principal component analysis. We assessed women’s empowerment in our study using 11 indicators common to both survey rounds. Principal component analysis applied to these indicators led to three different underlying factors which we have named as: mother’s bargaining power (first component); mother’s autonomy (second component); and mother’s lack of freedom or restriction of movement (third component). One indicator, whether the mother’s name was on the rental/property ownership document, didn’t load onto the three factors and was included as a separate covariate in our models. The set of variables grouped under each factor, their rotated factor loadings (pattern matrix) and unique variances have been made available as supplemental material.

### Statistical methods

As mentioned earlier, women’s empowerment measures were assessed using the Principal component analysis (PCA) technique applied to various indicators of autonomy/restriction, decision-making status and bargaining power. Since these indicators were all on a categorical scale as opposed to a continuous scale, PCA was based on estimated polychoric correlation coefficients among all selected indicators [20].

Women’s maternal and child health care services (MCH) utilization changes over time and the impact of insurance status were assessed using multilevel modelling approach, allowing us to consider the hierarchical and nesting structure in our data (clustering) [33]. Surveyed women in our data were nested within households, which in turn were nested within communities. Pregnant women living in the same communities are subjected to similar enabling factors or barriers that affect their utilization of maternal and child health care services than women living in other communities [20]. Similarly, communities were nested within districts, meaning that pregnant women living in the same districts would face similar policy and health care infrastructures than women living in other districts. Maternal and child health (MCH) related service delivery and policies (which are implemented through the Ministry of Health and Family Welfare) are mostly designed at the district and sub-district levels in India. Moreover, previous studies in the Indian context have found significant disparities in MCH service coverage and efficiency in service delivery across districts in India [34–36].

Given the ordered categorical nature of the response or dependent variable, we used multilevel mixed-effects ordered logistic regression models to estimate changes in MCH service utilization in India between 2005 and 2012 [37]. We have considered village (community entity) and district level variations in maternal and child health care utilization as random effects in the multilevel modelling. Our models also included a random effect at the women’s level to take account of changes in the behavior of the same mother across time (repeated measures).

The first step in our modelling process was to fit a null model to mothers’ MCH utilization ordinal scores with an intercept term and random effects at the district, village and mother levels, but without any explanatory variables (Model 0). Model 0 (or variance components model) allowed to estimate the various sources of variability (variance components: district, village and mother) and to partition the total variance into the four levels of hierarchy (district, village, mother and within mother’s repeated measures residual variance). Notice that, according to Steele [38], level 1 residual variance (which capture the within-mother differences between 2005 and 2011–12) is 1 for a probit model and 3.29 for a logit model.

Six subsequent models were fitted to the data, all including random effects at the district, village and mother levels. Model 1 included only wave or year of survey as a specific fixed-effect factor. Model 2 added to Model 1, health insurance status, as the main exposure factor, while Model 3 included the interaction between 'year of survey' and 'health insurance' to Model 2 to capture the differential due to health insurance in 2011–12 round compared to 2005. In Model 4, maternal empowerment variables were added into Model 3, while Model 5 added the interaction between 'year of survey' and 'maternal empowerment variables' to Model 4. The final model, Model 6, considered Model 5 (but with only those interaction terms between 'year of survey' and 'maternal empowerment variables which were significant) and added household and mother’s level covariates.

Estimated coefficients were reported along with associated 95% confidence interval. Log-likelihood and Wald Chi-square statistics were also reported. All the analyses were performed using Stata 16 [39].

### Descriptive statistics

Tables 1 and 2 below present the descriptive statistics of all variables. We can see that the optimal MCH utilization (all 3 indicators used) rose from 16.1% in 2005 to 24.8% in 2011/12 indicating that access to maternal and child health care services had increased over the 2005 – 2012 period. Similarly, the percentage of households who had access to health insurance increased from a mere 1.7 percent in 2005 to over 10 percent in 2011/12. The average household size and percentage of households holding Below Poverty Line (BPL) cards were similar across the two time periods. Nor was there much difference between the average years of schooling indicating that mothers hadn’t gone for additional years of schooling between 2005 and 2012.

**Table 1:** Descriptive statistics 2005

Variable	Mean/Proportion	S. D.	Min.	Max.
MCH utilization outcome				
*MCH* = *0 (none of indicators)*	0.369	0.482	0	1
*MCH* = *1 (only one indicator)*	0.243	0.429	0	1
*MCH* = *2 (only 2 indicators)*	0.228	0.420	0	1
*MCH* = *3 (all 3 indicators)*	0.161	0.367	0	1
Household has Insurance				
*No*	0.983	0.130	0	1
*Yes*	0.017	0.130	0	1
Household size	6.556	2.715	2	22
Below Poverty Line (BPL) status				
*No*	0.617	0.486	0	1
*Yes*	0.382	0.486	0	1
**Maternal characteristics**				
Mother’s Age (years)	25.489	4.938	15	48
Mother’s Education (years)	4.695	4.793	0	15
Total Children born	2.311	1.662	1	11
Health status of mother (%)				
*Good or very good*	0.692	0.461	0	1
*Ok*	0.272	0.445	0	1
*Poor or very poor*	0.035	0.184	0	1
**Indicators of MCH utilization**				
Place of Birth *(%)*				
*Non-Institutional delivery*	0.546	0.497	0	1
*Institutional delivery*	0.454	0.497	0	1
Antenatal visits (%)				
*Three or less*	0.606	0.489	0	1
*Four or more*	0.393	0.489	0	1
Postnatal visits (%)				
*No visits*	0.658	0.474	0	1
*Had visits*	0.341	0.474	0	1
**Maternal empowerment indicators**				
*Mother’s bargaining power*	0.139	0.254	-0.250	1.336
*Mother’s autonomy*	0.731	0.484	-0.214	1.421
*Mother’s restriction of movement*	-0.054	0.401	-0.844	1.420
Mothers’ name is on rental/ownership document				
*No*	0.908	0.289	0	1
*Yes*	0.092	0.289	0	1

**Table 2:** Descriptive statistics 2011/12

Variable	Mean/Proportion	S. D.	Min.	Max.
MCH utilization outcome				
*MCH* = *0 (none of indicators)*	0.119	0.324	0	1
*MCH* = *1 (only one indicator)*	0.301	0.459	0	1
*MCH* = *2 (only 2 indicators)*	0.332	0.471	0	1
*MCH* = *3 (all 3 indicators)*	0.248	0.431	0	1
Household has Insurance				
*No*	0.897	0.304	0	1
*Yes*	0.103	0.304	0	1
Household size	6.397	2.308	2	22
Below Poverty Line (BPL) status				
*No*	0.656	0.475	0	1
*Yes*	0.344	0.475	0	1
**Maternal characteristics**				
Mother’s Age (years)	32.106	4.701	20	56
Mother’s Education (years)	4.777	4.741	0	15
Total Children born	3.690	1.803	2	15
Health status of mother (%)				
*Good or very good*	0.792	0.406	0	1
*Ok*	0.134	0.341	0	1
*Poor or very poor*	0.074	0.262	0	1
**Indicators of MCH utilization**				
Place of Birth *(%)*				
*Non-Institutional delivery*	0.369	0.482	0	1
*Institutional delivery*	0.631	0.482	0	1
Antenatal visits (%)				
*Three or less*	0.532	0.499	0	1
*Four or more*	0.468	0.499	0	1
Postnatal visits (%)				
*No visits*	0.333	0.472	0	1
*Had visits*	0.666	0.472	0	1
**Maternal empowerment indicators**				
*Maternal bargaining power*	0.216	0.299	-0.240	1.345
*Maternal autonomy*	0.903	0.412	-0.127	1.324
*Maternal freedom of movement*	-0.170	0.314	-0.598	0.976
Mothers’ name is on rental/ownership document				
*No*	0.882	0.322	0	1
*Yes*	0.117	0.322	0	1

However, the proportion of institutional delivery had significantly increased from 45.4 percent in 2005 to 63.1 percent in 2012. The incidence of having four or more antenatal checkups had similarly gone up from 39.3 percent to approximately 46.8 percent during the study period. There was also a remarkable increase in the proportion who had received postnatal visits, which rose from 34.1 percent in 2005 to 66.6 percent in 2012.

The average scores for the maternal empowerment indicators, mother’s bargaining power and autonomy, had increased pointing out that mother’s socioeconomic status within the household had improved during the period. There was also a fall in mother’s restriction of movement showing improvement in mother’s freedom to go out of the house.

## Results

### District, community and mother sources of variation

As shown in Table 3, the likelihood ratio test statistic is highly significant showing that the four-level variance components multilevel ordered logistic regression model to mothers’ maternal and child health care utilization ordinal scores is a better fit to the data than the single-level model. Likelihood ratio tests further confirm that the four-level model is more preferred than three-level or two-level models.

**Table 3:** Null model with district, community and mother random effects

	**Coefficient (S.E.)**	**95% Confidence Interval**
Between District variance	0.393 (0.109)	(0.228, 0.677)
Between Community variance	0.389 (0.067)	(0.278, 0.545)
Between mother variance	1.312 (0.122)	(1.093, 1.575)
/cut 1	-1.410 (0.102)	(-1.610, -1.209)
/cut 2	0.197 (0.100)	(0.001, 0.393)
/cut 3	1.913 (0.105)	(1.706, 2.120)
Log likelihood = -9992.640		
LR test vs. ologit model: chi2(3) = 654.49		
Prob > chi2 = 0.0000		

Note: LR chi2(1) = 70,786.87 (Prob > chi2 = 0.0000) & LR chi2(1) = 87.86 (Prob > chi2 = 0.0000) are likelihood ratio test statistics from comparing the four level model with three level models with random effects at the community & mother levels, and random effects at the district and mother levels, respectively

Thus, the odds of mothers’ MCH utilization levels vary by district, community and mother over time and these variations needed to be considered in the analysis. Indeed, a decomposition of the total variance indicates that the between-district variance (level 4 residual variance) is estimated as 0.393, the within-district-between-villages variance (level 3 residual variance), on the other hand, is 0.389, while the within-villages-between-mothers variance (level 2 residual variance) is 1.312. Therefore, variance partition coefficients (VPC) showed that differences at the district level accounted for 7.3% of the total variation in MCH care utilization odds whereas differences at the community level accounted for 7.2% of the total variation. On the other hand, differences between mothers accounted for 24.4% of the variation, with the remaining 61.1% attributable to within mother differences across the two survey years. Thus, most of the variation in MCH care utilization was observed at the maternal level and our most comprehensive model below will control for maternal level predictors.

### Changes in maternal and child healthcare service utilization: impact of health insurance

Table 4 below presents results from estimation of six multilevel ordered logistic regression models with predictors, all allowing for random effects at the district, village and mother levels. The first column of Table 4 (Model 1) indicated that MCH utilization levels had significantly increased between 2005 and 2011–12. The odds of attaining a higher MCH utilization level increased by almost four-fold over the period of study. The second column (Model 2) showed that the odds of being in a higher MCH utilization level increased by 49% for those mothers from households with health insurance (either private or public). The third column (Model 3) enabled us to compare the impact of health insurance on MCH utilization levels before and after the publicly funded insurance scheme came into existence. The odds ratio associated to the interaction term indicated that the effect of health insurance on MCH service utilization in 2011–12 was 0.42 times weaker compared to 2005. In particular, our estimates showed that the odds of attaining higher MCH utilization levels increased by threefold for mothers who had (private) health insurance in 2005, whereas the corresponding figure for 2011–12 was 1.3 times.

**Table 4:** Multilevel ordered logistic regression with predictors

	**Model 1**	**Model 2**	**Model 3**	**Model 4**	**Model 5**	**Model 6**
**Variables**	**Odds Ratio** **(95% C.I.)**	**Odds Ratio** **(95% C.I.)**	**Odds Ratio** **(95% C.I.)**	**Odds Ratio** **(95% C.I.)**	**Odds Ratio** **(95% C.I.)**	**Odds Ratio** **(95% C.I.)**
**Wave**						
2005	Reference	Reference	Reference	Reference	Reference	Reference
2011–12	3.865^a^ (3.497, 4.272)	3.762^a^ (3.398, 4.165)	3.862^a^ (3.483, 4.285)	3.492^a^ (3.106, 3.928)	4.992^a^ (3.856, 6.464)	6.674^a^ (5.104, 8.727)
**Health Insurance**						
No		Reference	Reference	Reference	Reference	Reference
Yes		1.493^a^ (1.187, 1.878)	3.100^a^ (1.761, 5.454)	2.887^a^ (1.537, 5.423)	2.805^a^ (1.495, 5.264)	1.275 (0.668, 2.434)
**Wave # Health Insurance**			0.418^a^ (0.226, 0.774)	0.434^b^ (0.220, 0.858)	0.443^b^ (0.225,0.875)	0.886 (0.444, 1.767)
**Mothers age**						1.026^a^ (1.010, 1.043)
**Mothers education**						1.155^a^ (1.136, 1.175)
**Total Children born**						0.781^a^ (0.744, 0.822)
**Household size**						0.995 (0.970, 1.021)
**Consumption per capita Quintile**						
First quintile						Reference
Second quintile						1.289^a^ (1.085, 1.531)
Third quintile						1.718^a^ (1.430, 2.065)
Fourth quintile						2.324^a^ (1.911, 2.825)
Fifth quintile						2.529^a^ (2.027, 3.157)
**Mother’s health status**						
Good or very good						Reference
Ok						0.922 (0.794, 1.071)
Poor or very poor						1.028 (0.800, 1.322)
**Holds Below Poverty Line card**						
No						Reference
Yes						1.167^b^ (1.029, 1.324)
**Mother’s bargaining power (MBP)**				1.236 ^c^ (0.999, 1.529)	1.100 (0.793, 1.524)	1.172 (0.950, 1.446)
**Mother’s autonomy (MA)**				2.031^a^ (1.769, 2.332)	2.576 ^a^ (2.153, 3.081)	2.294^a^ (1.896, 2.776)
**Mother’s restriction on movement (MRM)**				0.791^a^ (0.667, 0.938)	0.987 (0.799, 1.219)	1.020 (0.814, 1.279)
**Wife’s name on rental/property document**						
No				Reference	Reference	Reference
Yes				1.449^a^ (1.196, 1.757)	1.276^c^ (0.955, 1.706)	1.276^b^ (1.056, 1.542)
**Wave # MBP**					1.179 (0.773, 1.799)	
**Wave # MA**					0.557^a^ (0.427, 0.726)	0.507^a^ (0.389, 0.662)
**Wave # MRM**					0.517^a^ (0.367, 0.729)	0.676^b^ (0.482, 0.948)
**Wave # Wife’s name on rental/property**					1.184 (0.806, 1.738)	
**B/n District variance**	0.556 (0.326, 0.948)	0.570 (0.335, 0.970)	0.559 (0.329, 0.952)	0.527 (0.302, 0.918)	0.526 (0.302, 0.917)	0.136 (0.066, 0.283)
**B/n Community variance**	0.508 (0.362, 0.714)	0.508 (0.361, 0.715)	0.501 (0.355, 0.706)	0.443 (0.299, 0.655)	0.428 (0.288, 0.636)	0.109 (0.051, 0.231)
**B/n Mother variance**	2.167 (1.868, 2.515)	2.179 (1.878, 2.530)	2.165 (1.864, 2.514)	2.158 (1.816, 2.564)	2.101 (1.764, 2.503)	1.019 (0.773, 1.344)
**/cut 1**	-0.927^a^ (-1.164, -0.690)	-0.917^a^ (-1.156, -0.678)	-0.904^a^ (-1.142,, -0.667)	-0.283^a^ (-0.541, -0.026)	-0.144 (-0.416, 0.127)	0.804^a^ (0.335, 1.272)
**/cut 2**	0.955^a^ (0.717, 1.192)	0.971^a^ (0.731, 1.211)	0.982 ^a^ ( 0.744, 1.221)	1.617^a^ (1.355, 1.879)	1.752^a^ (1.476, 2.029)	2.706^a^ (2.229, 3.183)
**/cut 3**	2.912^a^ (2.657, 3.167)	2.934^a^ (2.677, 3.191)	2.943^a^ (2.688, 3.199)	3.633^a^ (3.346, 3.921)	3.763^a^ (3.463, 4.063)	4.722^a^ (4.220, 5.224)
**Log likelihood**	-9602.158	-9566.514	-9562.649	-8275.384	-8261.028	-6839.440
**Wald chi2(1)**	701.43	711.09	717.95	739.48	761.28	180.94
**Prob > chi2**	0.000	0.000	0.000	0.000	0.000	0.000

Notes: ^a^, ^b^ and ^c^ refer to significant effect at the 1 percent, 5 percent and 10 percent levels of significance respectively

Results from Model 4 showed that three of women’s measures—mother’s autonomy, restriction on mother’s movement and the mother having her name on property documents – were highly significant correlates of MCH service utilization. The results from the interactions ‘year of survey’ with ‘women’s empowerment components’, in Model 5, indicated that the effect of maternal autonomy on increasing MCH utilization weakened significantly over the 2005 – 2011/12 period. Conversely, restriction on maternal movement was shown to have a much stronger and significant impact in reducing MCH utilization in 2011/12 than in 2005.

Results of the final model, Model 6, indicate that, after controlling for a host of confounders at the maternal and household levels, the year of survey (or wave) remained statistically significant, with mothers in 2011–12 found to have a significantly higher odds of MCH service utilization levels than mothers in 2005. However, the odds ratios associated to health insurance (health insurance effect in 2005) and the differential impact (interaction term) were no longer statistically significant.

Various covariates measured at the maternal and household level impacted mother’s MCH service utilization levels over the survey waves. Among these covariates, mother’s age was a significant correlate positively associated with MCH service utilization. Maternal education was also significant with mothers who have received more years of schooling more likely to utilize MCH services. On the other hand, total children born was negatively associated with MCH utilization, decreasing the odds by 22 percent.

Moreover, household consumption per capita was significant, the effect increasing with the quintile levels with mothers coming from higher quintiles more likely to utilize MCH services than mothers in the bottom quintile. Mothers from households that hold below poverty line cards (BPL) were also found to be more likely to access MCH services than those who do not possess BPL cards. Mother’s health status, however, was not a significant correlate of MCH care utilization. We also considered mothers’ past experience with miscarriage and/or still birth as a potentially significant confounder, but it didn’t represent any statistically significant influence on the outcome variable in this analysis.

Lastly, results showed that, as we moved from Model 1 to Model 6, variance components decreased as we added more and more covariates, especially in Model 6, with the addition of household and mother’s level predictors. Model 6 results suggested that these covariates play an important role in explaining all the three sources of variability in the ordered log-odds of MCH service utilization.

## Discussion

We investigated women’s maternal and child healthcare (MCH) service utilization behavior and assessed the factors affecting the odds of being in high levels of service utilization in India over the 2005 – 2012 period. All our models consistently indicated that mothers were more likely to access a wider set of MCH services in 2011–12 than 2005. This result is consistent with the various government initiatives and overall concerted efforts to increase pregnant women’s access to quality maternal health services in India since 2005 [10, 40]. Literature supports that institutional birth rates, for example, had sharply increased from 38% in 2005 to 74% in 2013 and this success was attributed to the conditional cash transfer program (JSY) launched in 2005 [7, 9, 41]. It was argued that the cash incentive offered under the program may have incentivized more women to take up institutional births [42, 43]. However, studies have established that JSY entitlements were not enough to cover out-of-pocket expenditures related to childbirth itself [8, 12].

The increased utilization of MCH services over our study period (2005—2012) may reflect changing attitudes among women (and Indian society) about the importance of professional care during pregnancy. A socio-cultural preference for home-based childbirth and viewing childbirth as a natural occurrence not needing professional assistance have been prevalent in India [8]. Garcia-Prado (p.96) [44] notes that “With respect to demand, the biggest challenge is to change behavioral patterns related to maternity and childbirth and promote the use of health services that can reduce maternal and neonatal deaths”. There is evidence that Accredited Social Health Activists (ASHAs), who provide counselling to pregnant women and arrange for their care, have helped to change the society’s attitude towards professional maternity care. Sidney et.al. [43] conducted in-depth interviews with women who had delivered in Madhya Pradesh and found that social norms about institutional births were changing in India and that pressure from ASHAs had helped to shape pregnant women’s and societies’ attitudes.

Overall, access to health insurance was shown to positively and significantly affect MCH service utilization in both 2005 and 2011–12 rounds. Health insurance is expected to reduce the burden of costs associated to institutional births, ante- and post- natal services, encouraging more mothers to access MCH services [10]. However, the negative differential impact, between 2005 and 2011–2012 rounds, indicated that the public insurance scheme (RSBY) (prevalent in 2011–12) didn’t have as strong impact as privately available insurance in 2005 in encouraging women to access MCH services. This means that the marginal increase in MCH utilization among those benefitting from the publicly available insurance scheme (2011/12) was less compared to those accessing private insurance (2005).

Studies have found that RSBY had not provided poor Indian families with any significant protection from financial risks and that families were still incurring significant out-of-pocket expenses for inpatient hospital care [45]. More worryingly, there is evidence that the financial burden on disadvantaged groups such as scheduled castes and tribes had increased and that there was little to no change in their utilization of health services [13, 46]. The increase in out-of-pocket expenses may likely be a result of enrolled households utilizing hospital services either not covered by RSBY or beyond the stipulated RSBY cap [47]. It has also been pointed out that some hospitals requested patients to purchase expensive medicines from elsewhere [48]. The RSBY scheme has also been criticized for its poor strategies to targeting poor families noting that the BPL list which is used to enroll families into the program is notorious for excluding disadvantaged groups such as scheduled tribes, scheduled casts, agricultural laborers and landless households [46].

Our results indicated that health insurance marginal effects decreased and lost their statistical significance when adjusting for household and mother level confounders, such as mother’s age, education and household wealth quintile (Model 6), suggesting that these covariates were more significant in determining access to MCH services over time than health insurance in India. Indeed, our finding that more educational attainment leads to more utilization of MCH services is supported by other studies in the literature (see for instance, [4, 49, 50]). Education increases mothers’ awareness of health-related information which makes them more predisposed to access MCH services [51]. Ali and Chauhan [50] also noted that “educational attainment is critical in imparting the feelings of self-worth and self-confidence which are critical in bringing the changes in health-related behavior” (p.9).

Moreover, our results indicated that mothers’ utilization of MCH services decreases with total children born, but not household size. As the number of children born to a mother increases, there would be more demand for the mother’s time at home adversely affecting her utilization of MCH services [52]. The lack of time, due to household chores and other responsibilities, was one of the factors Joshi et al. [53] identified as barriers to MCH utilization in Uttarakhand, India. Household wealth is another factor that has been identified in the literature as a significant correlate of MCH utilization [54, 55]. Our results indicated a clear increasing trend in the odds of reaching a higher MCH utilization level as we move from the lowest to the highest quintiles of consumption per capita. Mothers from lower consumption quintiles may face financial difficulties when trying to access MCH services. Although the government health care system is meant to provide maternal and child health care services free of charge, there are significant direct and indirect costs which include expenses on medicine, cost of transportation to and from public health facility, informal payments at the health facility (such as bribes) and forgone wages of husband and wife [5, 56]. These costs may push vulnerable and poor households into deeper poverty. Indeed, Zodpey and Farooqui [57] noted that around 50 million households slip into poverty each year due to out-of-pocket health expenditures.

Interestingly, our results also showed that households holding BPL cards were more likely to access MCH services compared to non-BPL households. Our data shows that only 15% of households who held BPL cards had availed themselves of health insurance in 2012 compared to a mere 1% in 2005. This indicates that more households had enrolled for health insurance after the public insurance scheme became available but that RSBY was not benefiting many poor households as it had been intended validating concerns about its poor targeting. We re-fitted the model by interacting ‘year of survey’ with BPL (result not reported here) and found that BPL was not a significant correlate of MCH utilization in 2005 but in 2011/12. This shows that BPL status significantly increased access to MCH service utilization in 2011–12 after the government covered this vulnerable group with a public insurance scheme [58].

Among variables that capture the empowerment of mothers in the household, results showed that the index of “maternal autonomy” was associated with more utilization of MCH services. This variable reflects the mother’s self-determination and power to make her own decisions. This may include her capacity to go out of the house on her own and complete tasks (such as visiting health centers) without assistance. The wider literature attributes higher maternal mobility to greater decision-making ability within the household [20, 59]. A mother who can go out and move freely is more likely to come across valuable information and advice on various aspects of maternal and child health in addition to being able to visit health centers for necessary health checks including antenatal and postnatal checkups [60]. Bloom et al. [18] had similarly established that women with greater freedom of movement were more likely to access maternal health care in North India. Mohanty and Gebremedhin [20] had also found that the marginal effects of the maternal autonomy indicators on birth registration varied across districts in India.

Having a mother with her name on the rental/ownership document of their residential property was also a significant correlate increasing mother’s utilization of MCH services. This variable captured economic empowerment and economically empowered women are more likely to make decisions on health care and other aspects of life that would benefit them and their children. Other studies in the literature have similarly established that women with more autonomy and decision-making capacity are more likely to access MCH services [61, 62].

## Strengths and Limitations

### Strengths

The publicly funded health insurance scheme (RSBY) covers all expenses related to hospital delivery, but there is lack of studies investigating its effect on institutional delivery in India. Most studies in the literature have focused on the impact of RSBY on out-of-pocket expenses and whether the scheme has been successful in preventing households from incurring catastrophic health expenditures. On the other hand, several studies have looked at the impact of JSY, the cash transfer, on maternal care. Another strength of the study is that it matches mothers whose delivery had been captured by two rounds of IHDS data allowing to capture changes over time, whereas most other studies use a cross-section of mothers whose delivery had been recorded at one point in time. The use of multilevel models is another strength of the study because our analysis takes into account clustering at the district, village and individual mother levels.

### Limitations

We were not able to identify from the IHDS data which women had received JSY cash transfer for delivery. Nor were we able to determine from the data if those accessing antenatal and postnatal services had been assisted by ASAHs as part of the JSY program. As a result, we were not able to explicitly control for JSY in our regression models, although the time fixed-effect included in our model would have captured some of the effects of the conditional cash transfer program. In addition, we were not able to take into account the cost of delivery and other costs associated with maternal and childcare because the data did not contain such information.

## Conclusion

We studied the factors associated with the utilization of maternal and child health care services in India over the 2005 – 2011/12 period, with special emphasis on the effect of a publicly funded health insurance scheme (RSBY) and specific maternal empowerment measures. Our models consistently showed that women were more likely to utilize MCH services in 2011/12 compared to 2005. This is most likely the result of change in women’s and societal attitude towards maternal care from one that viewed delivery as a natural event that didn’t require the assistance of a skilled birth attendant to one that increasingly recognized the importance of professional care for the health and safety of both mother and child.

Our results also indicated that access to health insurance increased MCH utilization, although its effect was no longer statistically significant when we controlled for other confounding factors such as maternal education and household wealth in our final model. Interestingly, we found that the publicly funded health insurance scheme (RSBY) was less effective in increasing MCH utilization compared to private insurance. Given the evidence that the public insurance scheme has failed to protect households from high cost of hospitalization, the scheme may need to be overhauled to increase the level of coverage and reduce costs of health care. Maternal empowerment indicators were also found to be significant correlates of MCH utilization in India. In particular, mother’s autonomy (capturing her ability to leave the house and complete tasks) and whether the mother’s name is on the rental or property ownership documents (capturing mother’s economic empowerment) increased the odds of high levels of MCH utilization. This points to the need to take maternal autonomy and empowerment into consideration when designing programmes and policies that aim to improve health services for women. Other significant correlates of MCH utilization in our study included maternal education, household wealth quintile and number of children born to the mother.

## Supplementary Information

**Additional file 1:** **Table 1.** Mother’s bargaining power, autonomy, and restriction of movement: Rotated factor loadings (pattern matrix) and unique variances – 2005 round. **Table 2.** Mother’s bargaining power, autonomy, and restriction of movement: Rotated factor loadings (pattern matrix) and unique variances – 2011/12 round. **Table 3.** Multilevel ordered logistic regression (controlling for past history of mishaps).

## Data Availability

The data underlying this study are third party and were collected by the India Human Development Survey (2005 and 2011–2012). We obtained the raw survey data from Data Sharing for Demographic Research (DSDR) website. These data are available at the following link:http://www.icpsr.umich.edu/icpsrweb/DSDR/studies/36151#datasetsSection. The authors confirm that others would be able to access the data in the same manner and that the authors did not have any special access privileges that others would not have.
