# Supplementary material for: Public health insurance and maternal health care utilization in india: evidence from the 2005–2012 mothers’ cohort data
Source: BMC Pregnancy Childbirth. 2022 Feb 25;22:155. doi: 10.1186/s12884-022-04441-4 (PMC8876067; doi:10.1186/s12884-022-04441-4)
Supplement: Supplementary file 1 — Additional file 1: Table 1. Mother’s bargaining power, autonomy, and restriction of movement: Rotated factor loadings (pattern matrix) and unique variances – 2005 round. Table 2. Mother’s bargaining power, autonomy, and restriction of movement: Rotated factor loadings (pattern matrix) and unique variances – 2011/12 round. Table 3. Multilevel ordered logistic regression (controlling for past history of mishaps). [file 12884_2022_4441_MOESM1_ESM.docx]

Supplemental material:

The rotated factor loadings from our principal component analysis are presented below. One maternal empowerment indicator – whether the mother’s name was on property or rental document – didn’t load on any of the factors and was included in our regressions separately.

Table 1: Mother’s bargaining power, autonomy, and restriction of movement: Rotated factor loadings (pattern matrix) and unique variances – 2005 round

| Variables | Factor 1: Mother’s bargaining power | Factor 2: Mother’s autonomy | Factor 3: Mother’s restriction of movement | Unique variation in variables |
| --- | --- | --- | --- | --- |
| 1. Mother has most say or decides jointly on what to cook on a daily basis | 0.511 | 0.321 | 0.148 | 0.614 |
| 2. Mother has most say or decides jointly on purchasing expensive item | 0.850 | 0.101 | 0.159 | 0.243 |
| 3. Mother has most say or decides on number of children she has | 0.771 | -0.007 | -0.045 | 0.403 |
| 4. Mother has most say or decides jointly on what to do if a child falls sick | 0.759 | 0.191 | 0.153 | 0.364 |
| 5. Mother has most say or decides jointly to whom her children should marry | 0.861 | -0.015 | 0.181 | 0.226 |
| 6. Mother can visit Health Centre alone | 0.102 | 0.556 | -0.258 | 0.614 |
| 7. Mother can visit relative/friend alone | 0.072 | 0.883 | 0.281 | 0.136 |
| 8. Mother goes out with husband for meals, cinema | 0.049 | 0.849 | 0.286 | 0.194 |
| 9. Mother does not need permission/must inform to visit Health Centre | 0.204 | 0.281 | 0.865 | 0.131 |
| 10. Mother does not need permission/must inform to visit relative/friend | 0.085 | 0.161 | 0.931 | 0.100 |

Note: We have used Factor analysis with method: principal-component factors, rotation: orthogonal varimax (Kaiser off) and have retained 3 factors.

Table 2: Mother’s bargaining power, autonomy, and restriction of movement: Rotated factor loadings (pattern matrix) and unique variances – 2011/12 round

| Variables | Factor 1: Mother’s bargaining power | Factor 2: Mother’s autonomy | Factor 3: Mother’s restriction of movement | Unique variation in variables |
| --- | --- | --- | --- | --- |
| 1. Mother has most say or decides jointly on what to cook on a daily basis | 0.528 | 0.098 | 0.163 | 0.685 |
| 2. Mother has most say or decides jointly on purchasing expensive item | 0.832 | 0.103 | 0.104 | 0.287 |
| 3. Mother has most say or decides on number of children she has | 0.753 | 0.114 | 0.008 | 0.420 |
| 4. Mother has most say or decides jointly on what to do if a child falls sick | 0.822 | 0.127 | 0.143 | 0.288 |
| 5. Mother has most say or decides jointly to whom her children should marry | 0.852 | 0.021 | 0.163 | 0.247 |
| 6. Mother can visit Health Centre alone | 0.044 | 0.507 | -0.509 | 0.481 |
| 7. Mother can visit relative/friend alone | 0.118 | 0.875 | 0.204 | 0.179 |
| 8. Mother goes out with husband for meals, cinema | 0.079 | 0.870 | 0.257 | 0.171 |
| 9. Mother does not need permission/must inform to visit Health Centre | 0.268 | 0.225 | 0.839 | 0.174 |
| 10. Mother does not need permission/must inform to visit relative/friend | 0.057 | 0.259 | 0.876 | 0.163 |

Note: We have used Factor analysis with method: principal-component factors, rotation: orthogonal varimax (Kaiser off) and have retained 3 factors.

2. We considered mothers’ past experience with miscarriage and/or still birth as a potentially significant confounder, but it did not represent any statistically significant influence on the outcome variable in this analysis.

Table 3: Multilevel ordered logistic regression (controlling for past history of mishaps)

|  | Model 6 with mishap dummy & interaction | Model 6 with number of mishaps & interaction |
| --- | --- | --- |
| Variables | Odds Ratio  (95% C.I.) | Odds Ratio  (95% C.I.) |
| Wave |  |  |
| 2005 | Reference | Reference |
| 2011-12 | 6.941***  (5.246 , 9.183) | 6.915***  (5.231, 9.141) |
| Health Insurance |  |  |
| No | Reference | Reference |
| Yes | 1.285  (0.673 , 2.454) | 1.285  (0.673 , 2.455) |
| Wave*Health Insurance | 0.892  (0.447 , 1.781) | 0.8923  (0.447 , 1.782) |
| Mishap |  |  |
| No | Reference |  |
| Yes | 1.031  (0.806, 1.320) |  |
| No of Mishaps |  | 1.019  (0.868, 1.194) |
| Mishap*wave | 1.014  (0.750, 1.370) | 1.014  (0.846, 1.216) |
| Mothers age | 1.025***  (1.009 , 1.042) | 1.025***  (1.009 , 1.042) |
| Mothers education | 1.157***  (1.137, 1.177) | 1.157***  (1.137, 1.177) |
| Total Children born | 0.780***  (0.742 , 0.821) | 0.780***  (0.742 , 0.821) |
| Household size | 1.000  (0.975 , 1.026) | 1.000  (0.975 , 1.027) |
| Consumption per capita Quintile |  |  |
| First quintile | Reference | Reference |
| Second quintile | 1.315***  (1.104, 1.567) | 1.316***  (1.104, 1.567) |
| Third quintile | 1.775***  (1.473 , 2.139) | 1.774***  (1.472 , 2.138) |
| Fourth quintile | 2.345***  (1.921 , 2.861) | 2.344***  (1.921 , 2.861) |
| Fifth quintile | 2.509***  (2.001 , 3.145) | 2.507***  (2.000 , 3.143) |
| Mother’s health status |  |  |
| Good or very good | Reference | Reference |
| Ok | 0.942  (0.808 , 1.097) | 0.941  (0.808 , 1.097) |
| Poor or very poor | 1.028  (0.796, 1.327) | 1.026  (0.795, 1.324) |
| Holds Below Poverty Line card |  |  |
| No | Reference | Reference |
| Yes | 1.114**  (1.006, 1.302) | 1.115**  (1.006, 1.302) |
| Mother’s bargaining power (MBP) | 1.170  (0.944 , 1.449) | 1.169  (0.944 , 1.449) |
| Mother’s autonomy (MA) | 2.322***  (1.914 , 2.818) | 2.322***  (1.914 , 2.818) |
| Mother’s restriction on movement (MRM) | 1.072  (0.850 , 1.351) | 1.072  (0.851 , 1.352) |
| Wife’s name on rental/property document |  |  |
| No | Reference | Reference |
| Yes | 1.305**  (1.076 , 1.582) | 1.304**  (1.076 , 1.582) |
| Wave *MBP |  |  |
| Wave *MA | 0.496***  (0.378 , 0.650) | 0.497***  (0.379 , 0.651) |
| Wave *MRM | 0.636**  (0.450 , 0.897) | 0.636**  (0.451 , 0.897) |
| Wave * Wife’s name on rental/property |  |  |
| B/n District variance | 0.136  (0.066 , 0.283) | 0.136  (0.066 , 0.283) |
| B/n Community var. | 0.109  (0.051 , 0.231) | 0.109  (0.051 , 0.231) |
| B/n mother variance | 1.019  (0.773 , 1.344) | 1.019  (0.773 , 1.344) |
| /cut 1 | 0.846***  (0.373, 1.320) | 0.845***  (0.372 , 1.318) |
| /cut 2 | 2.734***  (2.251 , 3.217) | 2.732***  (2.249 , 3.215) |
| /cut 3 | 4.771***  (4.261, 5.280) | 4.769***  (4.259 , 5.278) |
| Log likelihood | -6581.264 | -6581.154 |
| Wald chi2(3) | 165.04 | 165.01 |
| Prob > chi2 | 0.000 | 0.000 |
